# Plasma Homocysteine Is Associated with Increased Oxidative Stress and Antioxidant Enzyme Activity in Welders

**DOI:** 10.1155/2013/370487

**Published:** 2013-09-09

**Authors:** Hung-Hsin Liu, Tung-Sheng Shih, Hsin-Ru Huang, Shih-Chien Huang, Lien-Hsiung Lee, Yi-Chia Huang

**Affiliations:** ^1^Department of Occupational Medicine, School of Occupational Safety and Health, Chung Shan Medical University, Chung Shan Medical University Hospital, Taichung 40201, Taiwan; ^2^Graduate Institute of Environmental Health, College of Public Health, China Medical University and Hospital, Taichung 40402, Taiwan; ^3^Department of Nutrition, Mennonite Christian Hospital, Shoufeng Branch, Hualien 974, Taiwan; ^4^Department of Health and Nutrition Biotechnology, Asia University, Taichung 41354, Taiwan; ^5^Institute of Occupational Safety and Health, Council of Labor Affairs, Executive Yuan, Taipei 22143, Taiwan; ^6^Department of Nutrition, School of Nutrition, Chung Shan Medical University, Chung Shan Medical University Hospital, Taichung 40201, Taiwan

## Abstract

The purpose of this study was to examine the association of vitamin B_6 _ status and plasma homocysteine with oxidative stress and antioxidant capacities in welders. Workers were divided into either the welding exposure group (*n* = 57) or the nonexposure controls (*n* = 42) based on whether they were employed as welders. There were no significant differences in vitamin B_6 _ status and plasma homocysteine concentration between the welding exposure group and the nonexposure controls. The welding exposure group had significantly higher levels of oxidized low-density lipoprotein cholesterol and lower erythrocyte glutathione concentration and superoxide dismutase (SOD) activities when compared to nonexposure controls. Plasma pyridoxal 5′-phosphate concentration did not correlate with oxidative stress indicators or antioxidant capacities in either group. However, plasma homocysteine significantly correlated with total antioxidant capacity (TAC) (partial *r*_*s*_ = −0.34, *P* < 0.05) and erythrocyte SOD activities (partial *r*_*s*_ = 0.29, *P* < 0.05) after adjusting for potential confounders in the welding exposure group. In the welding exposure group, adequate vitamin B_6_ status was not associated with oxidative stress or antioxidant capacities. However, elevated plasma homocysteine seemed to be a major contributing factor to antioxidant capacities (TAC and erythrocyte SOD activities) in welders.

## 1. Introduction

Welders constitute a large work force in Taiwan and worldwide. Welding is a process of joining metals by melting and fusing, and this process generates fumes. The fumes contain many metals and toxic gases [1], which produce free radicals and cause lipid peroxidation [2–4] and further cause a variety of adverse health effects. Welders exposed to these metals and toxic gases during the welding process have been shown to be associated with increased oxidative stress and alterations in antioxidants or antioxidant capacities when compared to controls [5–9]. Fortunately, the human body has antioxidant enzyme and nutrient systems that protect it against free radical attacks. The main antioxidant enzymes responsible for controlling oxygen free radicals are superoxide dismutase (SOD), catalase, and glutathione-dependent enzymes [i.e., glutathione peroxidase (GPx), glutathione reductase, and glutathione *S*-transferase (GST)] [10]. In addition to antioxidant enzymes, major antioxidant nutrients include vitamin A, C, and E. Recently, the potent antioxidant ability of vitamin B_6_ has been recognized [11–18]. 

Pyridoxal 5′-phosphate (PLP), the physiologically active coenzyme form of vitamin B_6_, may play a crucial role in antioxidant mechanism. Although the exact antioxidant mechanism has not been confirmed yet, PLP may directly scavenge radicals and inhibit lipid peroxidation [11, 16, 19–21] or may indirectly play an antioxidant role through serving as coenzymes in the glutathione antioxidant defense system. Plasma PLP serves as a coenzyme in the transsulfuration pathway of homocysteine to cysteine. Cysteine synthesized by this pathway is an important contributor to glutathione synthesis. It would then be reasonable to hypothesize that greater oxidative stress might be associated with lower vitamin B_6_ status, a higher homocysteine concentration, and an impaired glutathione-dependent antioxidant defense system [22–24].

Welders are particularly susceptible to fume exposure during the welding process. In this high oxidative stress environment, the utilization and metabolic turnover of vitamin B_6_ increase, and this lowers the body's pool of the vitamin. Although the effects of welding fume exposures on oxidative stress and antioxidant capacities have been studies in welders, no data on the associations of vitamin B_6_ status and homocysteine with oxidative stress and antioxidant capacities have been reported. The purpose of this study was to examine the associations of vitamin B_6_ status (plasma and erythrocyte PLP) and plasma homocysteine with oxidative stress and antioxidant capacities in welders. 

## 2. Materials and Methods

### 2.1. Participants

This cross-sectional study enrolled workers from the industry (Changhua, Taiwan), which offers a complete line of innovative fitness products in central Taiwan. This study was approved by the Institutional Review Board of Chung Shan Medical University hospital (Taichung, Taiwan), and each participant signed the informed consent form.

Welders were recruited into the welding exposure group if they were older than 20 years and had been employed full time for at least 3 months. Welders did not work in a specific area but were involved in various welding-related processes, including formulating, mixing, loading, and welding application and were exposed to variable levels of fumes. The control participants were white-collar office workers employed in the same company who were not exposed to welding fumes, and they were assigned into the nonexposure control group. Exclusion criteria were pregnancy or lactation, illness, history of gastrointestinal disorder, cardiovascular disease, liver and renal diseases, diabetes, cancer, alcoholism, or other metabolic disease. 

All subjects' age, smoking status and drinking habits, welding exposure time, and duration of employment were recorded. Body weight and height were measured; the body mass index (BMI; kg/m^2^) was then calculated. Fasting venous blood specimens were collected in vacutainer tubes (Becton Dickinson, Rutherford, NJ, USA) containing EDTA as an anticoagulant or without anticoagulant and were centrifuged (2500 rpm, 15 min) to separate serum/plasma and red blood cells then analyzed immediately or stored frozen (−80°C) until analysis. Spot urine samples were collected from each participant. 

### 2.2. Biochemical Analyses

Hematological entities (i.e., albumin, hemoglobin, creatinine, triglycerides, total cholesterol, low-density lipoprotein cholesterol (LDL), and high-density lipoprotein cholesterol) were measured by using an automated biochemical analyzer. Plasma and erythrocyte PLP were determined by high performance liquid chromatography (HPLC) according to a method described by Talwar et al. [25]. The inter- and intra-assay variabilities were 4.38% (*n* = 11) and 1.23% (*n* = 5) for plasma PLP and 7.16% (*n* = 12) and 3.33% (*n* = 5) for erythrocyte PLP, respectively. A plasma PLP level ≥ 20 nmol/L has been suggested as an indicator of adequate vitamin B_6_ status [26, 27]. Plasma homocysteine was measured by using HPLC with a modified method as described previously [28]. Hyperhomocysteinemia was defined as a plasma homocysteine concentration ≥ 15 *μ*mol/L [29]. The inter- and intra-assay variabilities of plasma homocysteine were 4.03% (*n* = 8) and 1.72% (*n* = 5), respectively. Plasma lipid peroxidation was measured as the levels of malondialdehyde (MDA) according to a modified method as described by Lapenna et al. [30]. The MDA level was measured at an excitation wavelength of 515 nm and an emission wavelength of 555 nm using a fluorescence spectrophotometer. Oxidized LDL (ox-LDL) was measured with ox-LDL ELISA kit (Mercodia AB, Uppsala, Sweden). Among the methodologies used to evaluate total antioxidant capacity (TAC), the most widely used colorimetric method for serum and plasma samples are 2′-2′-azinobis-3-ethyl-benzothiazoline-6-sulfonate-based methods. Therefore, TAC was measured according to a method described by Erel [31], who developed a novel colorimetric and automated direct assay. Reduced glutathione concentration in erythrocyte was measured by using glutathione assay kit (Cayman Chemical Company, Michigan, USA). GPx catalyzes the reduction of hydroperoxides, including hydrogen peroxide, by reducing glutathione and functions to protect the cell from oxidative damage [32]. Erythrocyte GPx levels were measured by using GPx assay kit (Cayman Chemical Company, Ann Arbor, MI, USA). GST is a ubiquitous multifunctional enzyme, which plays a key role in cellular detoxification [32]. Erythrocyte GST was determined by using GST assay kit (Cayman Chemical Company, Ann Arbor, MI, USA). SOD catalyzes the dismutation of superoxide into oxygen and hydrogen peroxide, after which the peroxide can be destroyed by catalase and GPx. SOD is an important antioxidant defense in nearly all cells exposed to oxygen. Erythrocyte SOD was determined by using SOD assay kit (Cayman Chemical Company, Ann Arbor, MI, USA). Urinary creatinine and 8-oxo-7,8-dihydro-2′-deoxyguanosine (8-OHdG) levels were measured. Urinary 8-OHdG concentration is a biomarker of oxidative DNA damage and repair and was determined using a competitive enzyme-linked immunoassay (Genox Corporation, Baltimore, MD, USA).

### 2.3. Statistical Analysis

Data were analyzed using the SAS statistical software (version 9.2, Statistical Analysis System Institute, Inc., Cary, NC, USA). A sample size of 47 subjects would allow detecting at least as well significantly correlated (*r* = 0.4) between plasma PLP or homocysteine and antioxidant enzyme activities with 80% statistical power and a two-sided *α* level of less than 0.05. A Kolmogorov-Smirnov test was performed to test the normal distribution. Differences in participants' demographic characteristics and biochemical values were compared for significance using student *t*-test or Mann-Whitney Rank Sum test between groups. Chi-square test was used for the analysis of categorical variables. Spearman correlation coefficient (*r*_*s*_) was used to analyze the association of vitamin B_6_ status and plasma homocysteine with oxidative stress indicators and antioxidant capacities in the welding exposure group and nonexposure controls. Spearman partial correlation coefficient (partial *r*_*s*_) was further used to analyze the association of vitamin B_6_ status and plasma homocysteine with oxidative stress indicators and antioxidant capacities in the welding exposure group and nonexposure controls after adjusting for age, gender, serum albumin and creatinine, smoking and drinking status, and duration of employment. Results were considered statistically significant at *P* < 0.05. Values presented in the text are means standard deviation (SD).

## 3. Results

Characteristics of participants are shown in Table 1. Ninety-nine subjects completed this study. Subjects' ages ranged from 20 to 61 years, with a mean age of 33.4 years. Fifty-seven welders were classified into the welding exposure group, while there were 42 white-collar office workers in the nonexposure control group. There were no significant differences in age, weight, height, BMI, serum triglycerides, total cholesterol, and LDL concentrations between the two groups. Welders had significantly higher levels of serum hemoglobin and creatinine and higher percentage of smoking and drinking habits when compared with the controls.

Table 2 shows subjects' vitamin B_6_ status, homocysteine, oxidative stress, and antioxidant enzyme activities during the study. Welders and nonexposure controls had similar vitamin B_6_ status (i.e., plasma and erythrocyte PLP), and none of welders or controls had deficient vitamin B_6_ status (plasma PLP < 20 nmol/L). No significant differences in plasma homocysteine, MDA and TAC levels, erythrocyte GPx and GST activities and urinary 8-OHdG concentration were observed between the two groups. However, welders had significantly higher ox-LDL levels and lower erythrocyte glutathione concentrations and erythrocyte SOD activities than did nonexposure controls. 

Plasma PLP strongly correlated with erythrocyte PLP in exposure welders (*r*_*s*_ = 0.68, *P* < 0.001) and nonexposure controls (*r*_*s*_ = 0.57, *P* < 0.001). The correlations between the mean of vitamin B_6_ status, plasma homocysteine, oxidative stress, and antioxidant capacities in welders and nonexposure controls are shown in Table 3. Plasma and erythrocyte PLP concentrations did not correlate with oxidative stress indicators (i.e., MDA, ox-LDL, and 8-OHdG) and antioxidant capacities (TAC, SOD, GPx, and GST activities) in welders. However, plasma homocysteine significantly positively correlated with the MDA level and erythrocyte SOD activity in welders. We further adjusted potential confounders including age, gender, serum albumin and creatinine, smoking and drinking status, and duration of employment which might affect oxidative stress and antioxidant capacities; plasma homocysteine still significantly positively correlated with erythrocyte SOD activity and negatively correlated with TAC in welders (Table 4). 

## 4. Discussion

It has been reported that welders exposed to welding fumes have higher oxidative stress and lower antioxidant capacities than that of controls [5–9]. Our welders had significantly higher ox-LDL concentrations and lower erythrocyte SOD activities when compared to controls. The finding indicates that welders are under greater oxidative stress and lower antioxidant capacity during welding process. In addition to oxidative stress indicators in plasma, we also measured urinary 8-OHdG concentration. Nuernberg et al. [9] observed that welders had a significantly higher rise in urinary 8-OHdG excretion when comparing preshift with postshift change. Urinary 8-OHdG concentration might normalize back to baseline by 24 hours from the start of the exposure [9]. Since we only collected urine sample from each subject at preshift time, this might explain why we did not observe the difference in urinary 8-OHdG concentration between welders and controls. 

In the past decades, vitamin B_6_ status and oxidative stress responses were mostly studied in animal models; very little data in humans have been reported. Recently, the association between higher oxidative stress and lower vitamin B_6_ status has been observed in older individuals [18], which might suggest the potent antioxidant ability of vitamin B_6_ in humans. Unfortunately, we did not observe the significant association of vitamin B_6_ status with oxidative stress indicators and antioxidant capacities in our subjects. Since none of our welders had inadequate vitamin B_6_ status (plasma PLP concentration < 20 nmol/L), other potential risk factors might affect welders' oxidative stress and antioxidant capacities. Although the exact role which the vitamin B_6_ compounds play as antioxidants is not clear yet, as long as welders maintain an adequate plasma PLP concentration, their vitamin B_6_ status is unlikely to affect their oxidative stress or antioxidant capacities. 

In the transsulfuration pathway of homocysteine metabolism, it requires plasma PLP as a coenzyme. Plasma homocysteine concentration, therefore, might be associated with vitamin B_6_ status. In addition to vitamin B_6_ status, plasma homocysteine concentration was measured in our subjects. Higher oxidative stress due to higher homocysteine concentration through homocysteine oxidation has been observed [33–35]. Elevated plasma homocysteine concentration may induce excessive production of reactive oxygen species and impair the glutathione-related antioxidant defense system thus leading to greater oxidative stress and lower antioxidant enzymatic activities [22, 36–38]. Our welders with higher homocysteine concentration had increased MDA level and erythrocyte SOD activities when potential confounders were not adjusted. Since smoking may be an important cause of oxidative stress and antioxidant capacities and this is known to be potentiated by exposure to fumes/toxic gases in the work place, smoking was forced into all models as a likely confounder. Our welders with higher homocysteine concentration were more likely to have lower TAC and higher erythrocyte SOD activity after potential confounders were adjusted. However, elevated homocysteine concentration has been found to be associated with decreased erythrocyte SOD activities in patients with cardiovascular heart diseases [39]. In a similar vein, Wilcken et al. [40] observed a strikingly positive relationship between excellular SOD and homocysteine in patients with homocystinuria. In agreement with the results of previous studies, elevated homocysteine concentration may cause the release of heparan sulfate-bound extracellular SOD into the blood [41] and thus constitute a protective mechanism with the effect of combating oxidative stress [40]. This would explain why our welders simultaneously had higher homocysteine concentration and increased SOD activity. Since we have observed that our welders with higher homocysteine concentration had lower TAC status, we could not rule out the possibility that welders with higher homocysteine concentration might have lower SOD activity if their welding exposure lasts for a longer period of time. It should be pointed out that this study had a cross-sectional design, so we could only observe the relationship between homocysteine and SOD activity at one point in time. Therefore, it was not possible to discriminate the short-term and long-term effects of elevated homocysteine concentration on antioxidant enzymatic activities in welders. 

There were some limitations in this study. Although we calculated the sample size to meet the statistical power criteria, a larger sample size might be needed to increase the significance of the associations between vitamin B_6_ and oxidative stress indicators and antioxidant capacities. The other limitation was that this was a cross-sectional design study, so the long-term associations of vitamin B_6_ and homocysteine with oxidative stress and antioxidant capacities in welders could not be assessed.

## 5. Conclusion

To the best of our knowledge, the present study is the first to show the associations of vitamin B_6_ status and homocysteine with oxidative stress indicators and antioxidant capacities in welders. The data herein indicate that, among welders, adequate vitamin B_6_ status was not associated with oxidative stress or antioxidant capacities. In addition to vitamin B_6_ status, elevated plasma homocysteine seemed to be a major contributing factor in relation to decreased TAC and increased erythrocyte SOD activity in welders. Further research into the long-term association of vitamin B_6_ and homocysteine concentration with oxidative stress and antioxidant enzymatic activities during welding exposure is warranted.

## Figures and Tables

**Table 1:** Demographic and clinical characteristics of participants.

Characteristics	Welding exposure (*n* = 57)	Nonexposure controls (*n* = 42)
Age (y)	33.29 ± 10.40	33.67 ± 7.46
Gender (male/female)	46/11^a^	9/33^b^
Height (cm)	165.15 ± 8.19	162.96 ± 7.49
Weight (kg)	61.94 ± 16.13	57.78 ± 12.31
Body mass index (kg/m^2^)	22.64 ± 5.69	21.55 ± 3.23
Duration of employment (yr)	1.99 ± 2.03^a^	3.64 ± 3.83^b^
Welding exposure time (hr/d)	7.54 ± 3.14	—
Serum albumin (g/dL)	4.61 ± 0.24^a^	4.51 ± 0.23^b^
Serum hemoglobin (g/dL)	15.20 ± 1.22^a^	13.88 ± 1.51^b^
Serum creatinine (mg/dL)	0.92 ± 0.16^a^	0.81 ± 0.16^b^
Lipid profiles		
Triglycerides (mg/dL)	86.58 ± 55.91	72.07 ± 36.17
Total cholesterol (mg/dL)	167.60 ± 31.12	176.17 ± 28.02
High-density lipoprotein (mg/dL)	60.18 ± 12.97^a^	67.69 ± 14.54^b^
Low-density lipoprotein (mg/dL)	101.70 ± 29.14	102.95 ± 23.94
Smoking (*n*, %)	23 (40.35%)^a^	1 (2.38%)^b^
Drinking (*n*, %)	6 (10.53%)^a^	1 (2.38%)^b^

Values are means ± SD.

Values with different superscript letter are significantly different between two groups; *P* < 0.05.

**Table 2:** Vitamin B_6_ status, oxidative stress indicators, glutathione, and antioxidant capacity.

Indicators	Welding exposure (*n* = 57)	Nonexposure controls (*n* = 42)
Vitamin B_6_ status		
Plasma PLP (nmol/L)	75.48 ± 72.23	71.16 ± 91.77
<20 nmol/L (%)	0	0
Erythrocyte PLP (pmol/g Hb)	178.36 ± 191.65	238.29 ± 462.87
Plasma homocysteine (*μ*mol/L)	12.13 ± 4.14	12.78 ± 4.75
≥15 *μ*mol/L (*n*, %)	11 (19.30%)	9 (21.43%)
Oxidative stress indicators		
Malondialdehyde (*μ*M)	0.61 ± 0.13	0.62 ± 0.11
Oxidized low-density lipoprotein (mU/L)	30329.74 ± 7507.95^a^	27521.15 ± 5891.29^b^
8-oxo-7,8-dihydro-2′-deoxyguanosine (ng/mg creatinine)	2.93 ± 1.21	3.03 ± 1.47
Antioxidant capacities		
Total antioxidant capacity (*μ*mol/L)	4278.05 ± 322.82	4280.41 ± 246.07
Erythrocyte glutathione (*μ*mol/g Hb)	0.65 ± 0.29^a^	0.73 ± 0.38^b^
Erythrocyte SOD (U/g Hb)	8461.28 ± 3202.72^a^	13391.24 ± 4729.43^b^
Erythrocyte GPx (nmol/min/g Hb)	61858.44 ± 15727.25	59021.55 ± 16498.69
Erythrocyte GST (nmol/min/g Hb)	10306.87 ± 6537.18	10943.85 ± 4201.24

Values are means ± SD. PLP: pyridoxal 5′-phosphate; Hb: hemoglobin; SOD: superoxide dismutase; GPx: glutathione peroxidase; GST: glutathione *S*-transferase.

Values with different superscript letter are significantly different between two groups; *P* < 0.05.

**Table 3:** Spearman correlation of vitamin B_6_ status and homocysteine with each indicator of oxidative stress and antioxidant capacity.

	Plasma PLP (nmol/L)		Erythrocyte PLP (pmol/g Hb)		Plasma homocysteine (μmol/L)	
	r_s_					
	Welding exposure	Nonexposure controls	Welding exposure	Nonexposure controls	Welding exposure	Nonexposure controls
Plasma homocysteine (*μ*mol/L)	−0.26*	−0.27	−0.51^†^	−0.32*	—	—
Oxidative stress indicators						
Malondialdehyde (*μ*M)	0.14	0.15	−0.09	−0.06	0.32*	0.14
Oxidized low-density lipoprotein (mU/L)	0.04	0.13	−0.06	−0.06	0.14	−0.19
8-oxo-7,8-dihydro-2′-deoxyguanosine (ng/mg creatinine)	−0.05	0.06	−0.04	0.08	−0.14	0.09
Antioxidant capacities						
Total antioxidant capacity (*μ*M)	0.24	−0.02	0.08	−0.15	−0.18	0.23
Erythrocyte glutathione (*μ*mol/g Hb)	−0.04	0.04	0.11	−0.13	−0.09	0.36*
Superoxide dismutase (U/g Hb)	0.04	0.11	0.03	0.40**	0.34*	0.02
Glutathione peroxidase (nmol/min/g Hb)	−0.15	0.11	0.04	0.34*	−0.08	−0.23
Glutathione *S*-transferase (nmol/min/g Hb)	−0.11	−0.16	0.00	−0.15	−0.23	0.07

PLP: pyridoxal 5′-phosphate; Hb: hemoglobin. *P < 0.05. **P < 0.01. ^†^ *P* < 0.001.

**Table 4:** Spearman partial correlation of vitamin B_6_ status and homocysteine with each indicator of oxidative stress and antioxidant capacity.

	Plasma PLP (nmol/L)		Erythrocyte PLP (pmol/g Hb)		Plasma homocysteine (*μ*mol/L)	
	Partial *r*_*s*_					
	Welding exposure	Nonexposure controls	Welding exposure	Nonexposure controls	Welding exposure	Nonexposure controls
Plasma homocysteine (*μ*mol/L)	−0.44^†^	−0.38*	−0.58^†^	−0.18	—	—
Oxidative stress indicators						
Malondialdehyde (*μ*M)	0.03	0.13	−0.16	0.13	0.15	−0.04
Oxidized low-density lipoprotein (mU/L)	0.04	0.25	−0.06	0.06	−0.04	−0.38*
8-oxo-7,8-dihydro-2′-deoxyguanosine (ng/mg creatinine)	−0.04	0.03	−0.08	0.22	−0.13	0.14
Antioxidant capacities						
Total antioxidant capacity (*μ*M)	0.20	0.09	0.12	0.04	−0.34*	0.08
Erythrocyte glutathione (*μ*mol/g Hb)	−0.09	0.14	0.04	0.15	0.04	0.19
Superoxide dismutase (U/g Hb)	−0.13	0.10	−0.05	0.41*	0.29*	0.28
Glutathione peroxidase (nmol/min/g Hb)	−0.07	0.07	0.01	0.15	0.08	−0.05
Glutathione *S*-transferase (nmol/min/g Hb)	−0.14	−0.18	−0.01	−0.18	−0.11	0.06

PLP: pyridoxal 5′-phosphate; Hb: hemoglobin. Adjusting for age, gender, albumin, creatinine, smoking, drinking, and duration of employment. *P < 0.05. **P < 0.01. ^†^ *P* < 0.001.
